# The carboxylate “gripper” of the substrate is critical for C‐4 stereo‐inversion by UDP‐glucuronic acid 4‐epimerase

**DOI:** 10.1002/1873-3468.70070

**Published:** 2025-05-16

**Authors:** Annika J. E. Borg, Laura De Cnop, Bernd Nidetzky

**Affiliations:** ^1^ Institute of Biotechnology and Biochemical Engineering Graz University of Technology, NAWI Graz Austria; ^2^ Austrian Centre of Industrial Biotechnology (acib) Graz Austria

**Keywords:** epimerase, reductase, rotation, SDR, short‐chain dehydrogenase/reductase, stereo‐inversion, UDP‐4‐keto‐pentose, UDP‐glucuronic acid, UDP‐xylose

## Abstract

UDP‐glucuronic acid 4‐epimerase (UGAepi) catalyzes the NAD^+^‐dependent interconversion of UDP‐glucuronic acid (UDP‐GlcA) and UDP‐galacturonic acid (UDP‐GalA) through a mechanism involving C4‐oxidation, 4‐keto‐intermediate rotation, and subsequent reduction. Here, the functional significance of the substrate's carboxylate group in the epimerization process was investigated using UDP‐4‐keto‐pentose, an analogous intermediate that lacks a carboxylate moiety. Site‐directed mutations were introduced into UGAepi from *Bacillus cereus* (BcUGAepi) to increase substrate binding pocket flexibility, enabling the variant enzymes to accommodate UDP‐4‐keto‐pentose more efficiently than the wild‐type does. Although these BcUGAepi variants partially maintained nonstereospecific C4‐epimerization activity with UDP‐GlcA, they demonstrated fully stereospecific reduction of UDP‐4‐keto‐pentose to UDP‐xylose. These findings highlight the critical role of the carboxylate moiety as an essential element for epimerization in BcUGAepi, and elucidate the structural determinants of substrate specificity in UGAepis.

## Graphical abstract

UDP‐glucuronic acid 4‐epimerase (UGAepi) catalyzes NAD^+^‐dependent interconversion of UDP‐glucuronic acid (UDP‐GlcA) and UDP‐galacturonic acid (UDP‐GalA) via C4‐oxidation, 4‐keto‐intermediate rotation, and C4‐reduction. Here, Borg et al. examined the role of the substrate's carboxylate group in the enzymic mechanism by analyzing NADH‐dependent reduction of UDP‐4‐keto‐pentose. UGAepi variants with glycine substitutions in the substrate binding pocket showed dramatically enhanced activity with UDP‐4‐keto‐pentose.

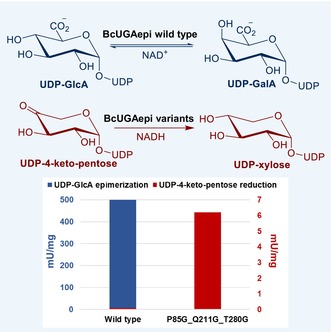

## Abbreviations

GALE, UDP‐galactose 4‐epimerase

SDR, short‐chain dehydrogenase/reductase

UDP‐GalA, UDP‐galacturonic acid

UDP‐GlcA, UDP‐glucuronic acid

UDP‐Xyl, UDP‐xylose

UGAepi, UDP‐glucuronic acid 4‐epimerase

**Fig. 1:**
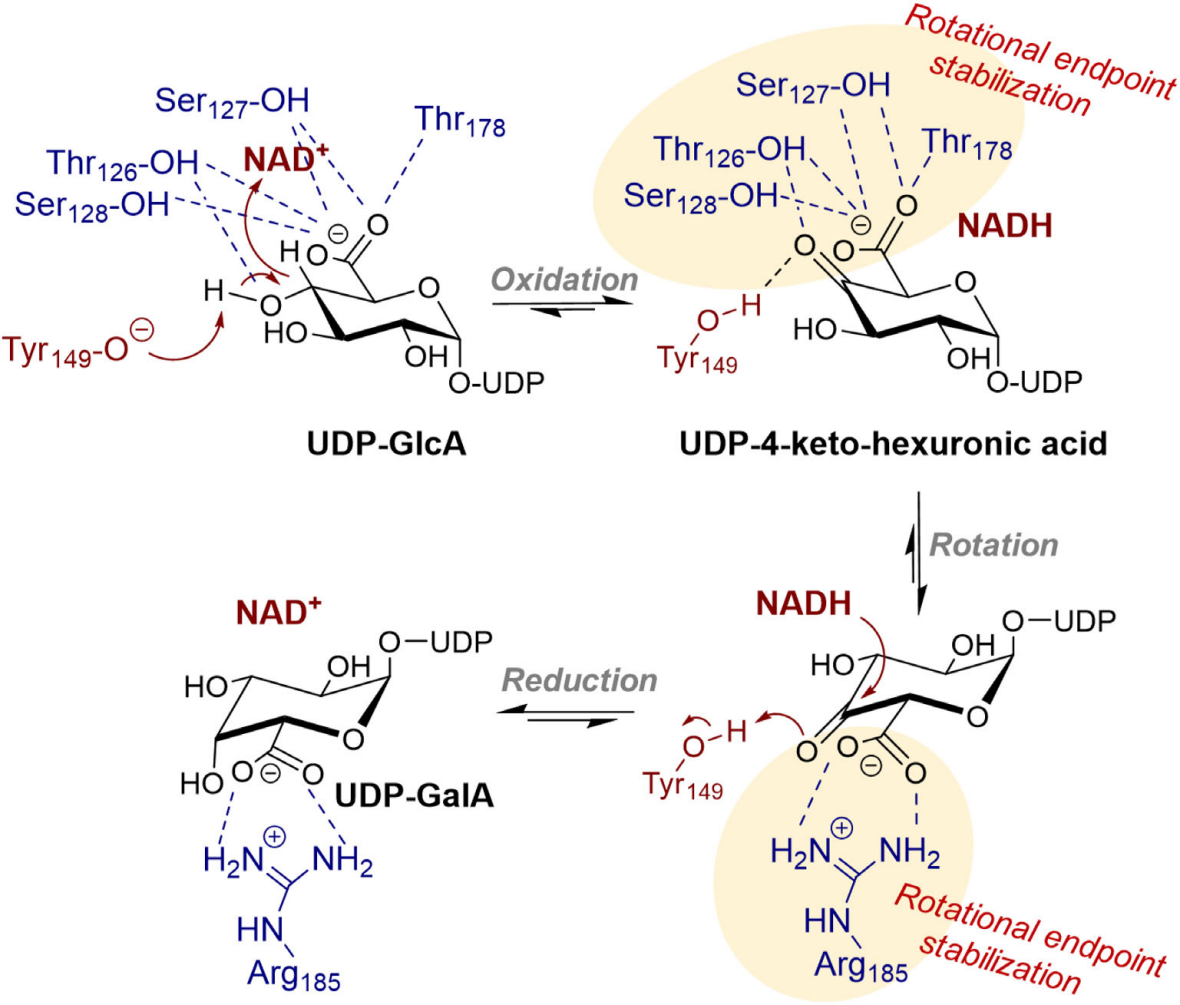
Proposed mechanism of BcUGAepi catalyzed interconversion of UDP‐glucuronic acid (UDP‐GlcA) and UDP‐galacturonic acid (UDP‐GalA). Binding pocket interactions in both endpoints of the UDP‐4‐keto‐hexuronic acid rotation hold the carboxylate group in an equatorial orientation, thus disfavoring the decarboxylation of the 4‐keto‐intermediate.

UDP‐glucuronic acid 4‐epimerase (UGAepi; EC5.1.3.6), a member of the short‐chain dehydrogenase/reductase (SDR) enzyme superfamily [1], mediates the NAD^+^‐dependent interconversion of UDP‐glucuronic acid (UDP‐GlcA) and UDP‐galacturonic acid (UDP‐GalA) via UDP‐4‐keto‐hexuronic acid intermediate (Fig. 1) [2, 3, 4, 5]. The reaction proceeds with the oxidation at UDP‐GlcA C4‐OH as the rate‐limiting step, followed by a rotation of the transient keto‐intermediate and fast NADH‐driven reduction to yield UDP‐GalA (Fig. 1) [2, 6]. Combination of structural, mutagenesis, and computational studies has delineated the critical role of rotational endpoint stabilization in the reaction of UGAepi from *Bacillus cereus* (BcUGAepi), emphasizing the stabilization of the carboxylate moiety in the rotational isomers [3, 6, 7]. In the substrate complex, the carboxylate of UDP‐GlcA is integrated within a tight hydrogen‐bonding network facilitated by threonine and serine residues (Fig. 1) [3]. Conversely, in the product complex, the carboxylate of UDP‐GalA is exclusively stabilized through ionic interactions with Arg185 (Fig. 1) [3]. Earlier work [6] has shown that disruption of this rotational endpoint stabilization via site‐directed mutagenesis yields enzyme variants that function as a primitive UDP‐xylose synthase, leading to UDP‐GlcA decarboxylation rather than epimerization. Furthermore, we have previously described the complete rotational trajectory of the 4‐keto‐intermediate, emphasizing the significance of stabilizing the carboxylate moiety in an equatorial orientation after rotation, to preclude the loss of the intermediate through decarboxylation [6, 7]. Nevertheless, the role of the carboxylate moiety in the actual epimerization process of UDP‐GlcA by UGAepi continues to be ambiguous. Structurally similar to UGAepi, UDP‐galactose 4‐epimerase (GALE) orchestrates a similar sequence of reaction steps (oxidation, rotation, and reduction; Fig. S1) to govern the conversion between UDP‐glucose (UDP‐Glc) and UDP‐galactose (UDP‐Gal) [8, 9, 10, 11].

**Fig. 2:**
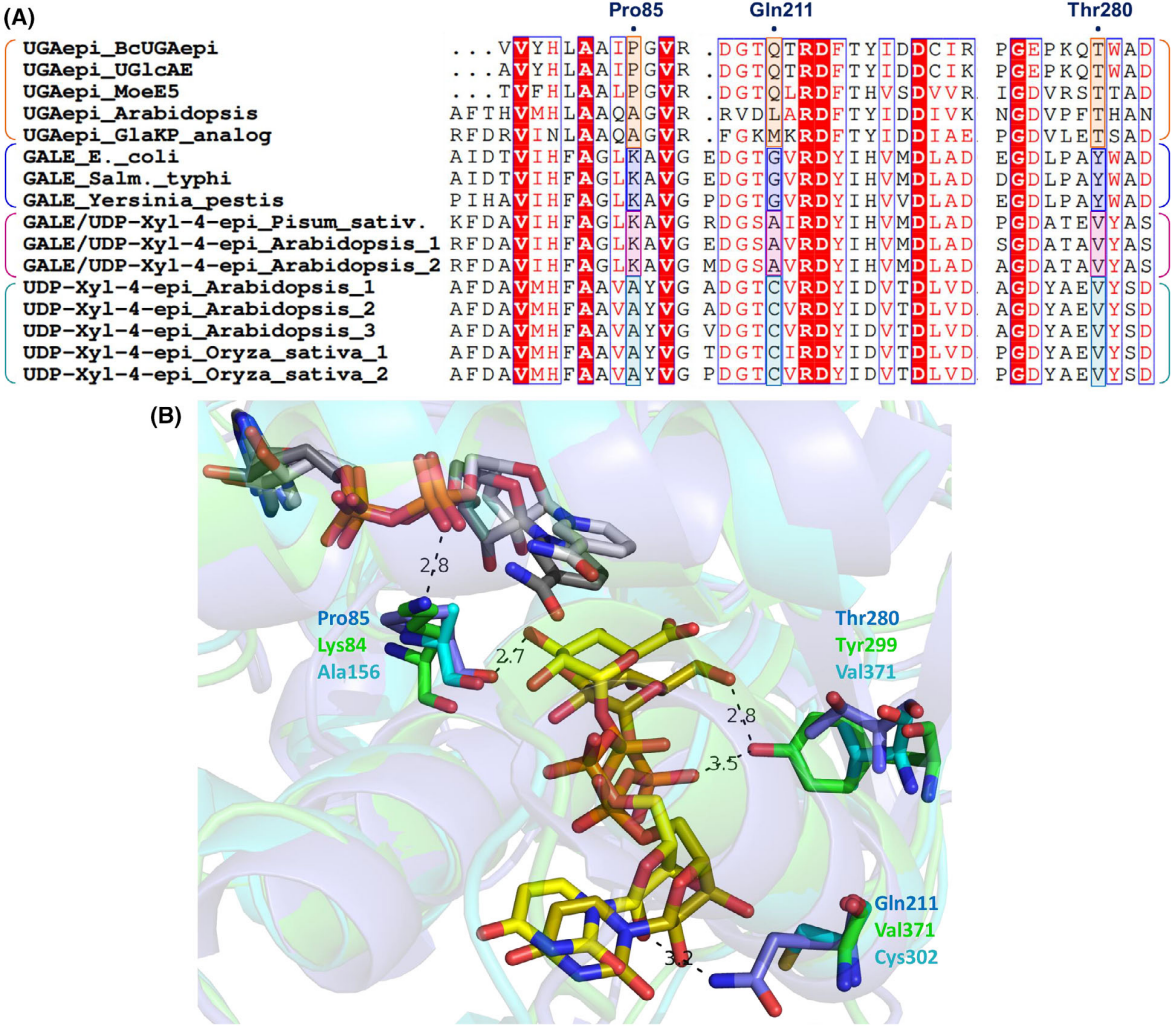
Sequence and structural comparison of UDP‐glucuronic acid 4‐epimerases (UGAepis), UDP‐galactose 4‐epimerases (GALEs) and UDP‐xylose 4‐epimerases. (A). Partial multiple sequence alignment of UGAepis (orange), GALEs (blue), bifunctional GALEs (dark pink) and UDP‐xylose 4‐epimerases (dark cyan) highlighting the residues chosen for mutagenesis in BcUGAepi (P85, Q211, T280). UniProt entries: UGAepi_BcUGApi (J8BY31), UGAepi_UGlcAE (A7GQD3), UGAepi_MoeE5 (A0A003), UGAepi_Arabidopsis (Q9M0B6), UGAepi_GlaKP_analog (Q9RP53), GALE_E.coli (P09147), GALE_Salm._typhi (Q56093), GALE_Yersinia_pestis (Q9F7D4), GALE/UDP‐Xyl‐4‐epi_Pisum_sativ. (B0M3E8), GALE/UDP‐Xyl‐4‐epi_Arabidopsis_1 (Q42605), GALE/UDP‐Xyl‐4‐epi_Arabidopsis_2 (Q8LDN8), UDP‐Xyl‐4‐epi_Arabidopsis_1 (Q9SA77), UDP‐Xyl‐4‐epi_Arabidopsis_2 (O64749), UDP‐Xyl‐4‐epi_Arabidopsis_3 (Q9SUN3), UDP‐Xyl‐4‐epi_Oryza_sativa_1 (Q8H930), UDP‐Xyl‐4‐epi_Oryza_sativa_2 (Q8H0B2). The sequence alignment was performed using MEGA X software and the visual representation of the aligned sequences generated using ESPript 3.0. (B) Active site close‐up of overlaid structures of BcUGAepi (blue; PDB: 6Z73; yellow carbons UDP‐GlcA, light gray carbons NAD^+^) [3], GALE from *E. coli* (green; PDB: 1XEL; dark yellow carbons UDP‐Glc, dark gray carbons NAD^+^) [10] and UDP‐xylose 4‐epimerase from *A. thaliana* (cyan, AlphaFold predicted structure, no ligands). The structural overlay was generated using pymol v4.6.

The substrate spectrum of UGAepis is typically confined to UDP‐GlcA/UDP‐GalA [4, 5, 12], with BcUGAepi demonstrating an inability to utilize UDP‐Glc/UDP‐Gal as a substrate [2]. The lack of activity toward UDP‐pentose substrates in UGAepis further proposes the essential role of the carboxylate moiety for epimerization. Here, we report BcUGAepi activity on UDP‐4‐keto‐pentose (UDP‐β‐l‐*threo*‐pentopyranosyl‐4‐ulose), a carboxylate‐lacking analog of UDP‐4‐keto‐hexuronic acid, upon introducing single or multiple site‐directed substitutions with glycine into the substrate binding pocket of the enzyme. Glycine was used to create more pocket space that might allow for higher flexibility in accommodating a different substrate besides UDP‐GlcA. The substituted positions (Pro85, Gln211, and Thr280) were chosen by sequence comparison of UGAepi to related 4‐epimerases that show different substrate specificity, such as GALE and UDP‐xylose 4‐epimerase (Fig. 2). The substitutions were distributed all over the binding pocket of BcUGAepi and are found in regions for binding of the GlcA sugar (Pro85) and the UDP nucleotide (Gln211, Thr280). The engineered single, double, and triple variants of BcUGAepi demonstrate a significant decrease (up to 250‐fold) in the activity toward nonstereospecific epimerization of UDP‐GlcA, and a correspondingly increased activity (up to ~70‐fold) toward the fully stereospecific, NADH‐dependent reduction in UDP‐4‐keto‐pentose yielding UDP‐xylose (UDP‐Xyl) as a single isomeric product. The change in reaction selectivity, that is, the activity ratio for UDP‐GlcA epimerization and UDP‐4‐keto‐pentose reduction, was significant (1.8 × 10^4^‐fold) between wild‐type enzyme (ratio: 5.6 × 10^3^) and P85G_Q211G_T280G triple variant (ratio: 0.32). Our study thus emphasizes the essential role of the substrate's carboxylate moiety, functioning as a rotational hinge necessary for the effective epimerization by BcUGAepi.

## Materials and methods

### Materials

The synthetic gene of BcUGAepi was ordered in pET17b‐expression vector (pET17b_BcUGAepi) from GenScript (USA). NAD^+^ (>98% purity) was from Roth (Karlsruhe, Germany). UDP‐d‐glucuronic acid (UDP‐GlcA; >98% purity) and sodium pyruvate were from Sigma‐Aldrich (Vienna, Austria). Deuterium oxide (99.96% ^2^H) was from Euriso‐Top (Saint‐Aubin Cedex, France). All other reagents and chemicals were of the highest available purity. GeneJET Plasmid Miniprep Kit (Thermo Scientific, Waltham, MA, USA) was used for plasmid DNA isolation. DpnI and Q5® High‐Fidelity DNA polymerase were from New England Biolabs (Frankfurt am Main, Germany) and d‐lactate dehydrogenase was from Megazyme (Vienna, Austria). All other enzymes were prepared in‐house. Oligonucleotide primers were from Sigma‐Aldrich (Vienna, Austria). *E. coli* NEB5α competent cells were from New England Biolabs (Frankfurt, Germany). *E. coli* Lemo21(DE3) cells were prepared in‐house.

### Enzymatic synthesis of UDP‐4‐keto‐pentose (UDP‐β‐l‐*threo*‐pentopyranosyl‐4‐ulose)

UDP‐4‐keto‐pentose was synthesized by converting UDP‐GlcA with the C‐terminal subunit of ArnA [13] from *E. coli* (expression and purification described elsewhere [14]). The reaction (20 mL) for the synthesis of UDP‐4‐keto‐pentose contained 2.0 mm UDP‐GlcA, 1.0 mm NAD^+^, 10 mm sodium pyruvate, 20 U·mL^−1^ d‐LDH, and 0.14 mg·mL^−1^ ArnA in 25 mm Tris buffer (1.0 mm DTT, pH 7.0). The reaction was performed at 30 °C without agitation and sampled at desired time points by quenching 5 μL of reaction mixture with 35 μL methanol prior to HPLC analysis. When UDP‐GlcA was completely converted to UDP‐4‐keto‐pentose (after ~20 h), the enzymes were removed by centrifugation (Vivaspin Turbo centrifugal filter tube, 30 kDa cut‐off, 21 130 g, 4 °C) and the supernatant was subjected to purification by anion exchange‐ and size‐exclusion chromatography based on a literature protocol [14].

### Bioinformatic analysis

Multiple sequence alignments were performed using the ClustalW program in MEGA X software [15], and the visual representation of the aligned sequences was generated using ESPript 3.0 [16]. Structural prediction of UDP‐xylose 4‐epimerase was obtained from AlphaFold DB [17, 18]. PyMOL v4.6 was used for the visualization of protein structures.

### Preparation of BcUGAepi variants

BcUGAepi variants were prepared using a modified QuikChange protocol, as described elsewhere [6]. PCRs were performed in a reaction volume of 50 μL using 20 ng of plasmid DNA as template and 0.2 μm of forward and reverse primer. Q5 DNA polymerase was used for DNA amplification. The sequences of DNA oligonucleotide primers used for mutagenesis in BcUGAepi are given in Table S1. Residual template DNA was removed by the addition of 10 U DpnI and incubation at 37 °C for 16 h. DpnI was precipitated at 80 °C for 20 min, the mixtures were centrifuged, and the PCR products analyzed by agarose gel electrophoresis and visualized by DNA staining. The PCR products were transformed into chemically competent *E. coli* NEB5α cells (New England Biolabs). Plasmid DNA was extracted and sequenced with T7prom/T7term primers provided by LGC Genomics (Berlin, Germany) to confirm the mutations. The correct constructs were transformed into *E. coli* Lemo21(DE3) cells, followed by expression and Strep‐tag affinity purification under standard conditions. A new Strep‐tag column was used for each variant to prevent contamination. The full details of expression and purification are reported elsewhere [19].

### Enzymatic reactions with BcUGAepi variants

**Fig. 3:**
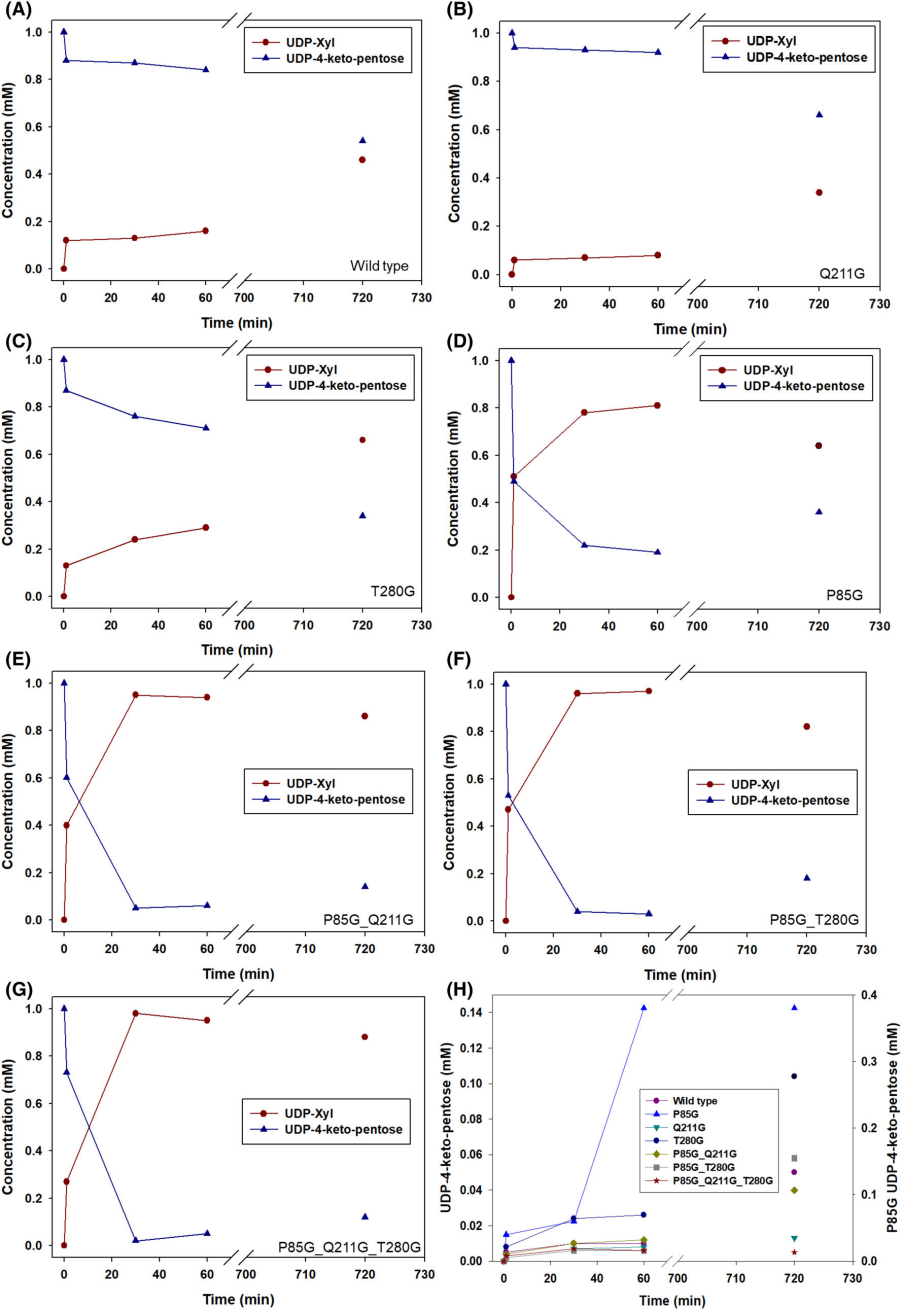
Time‐course analysis of the BcUGAepi variants (216 μm; 8.0 mg·mL^−1^) reacted with 1.0 mm of UDP‐4‐keto‐pentose or UDP‐xylose (UDP‐Xyl). The reactions of UDP‐4‐keto‐pentose (A–G) were performed with the addition of 10 mm NADH, and the reactions of UDP‐Xyl (H) with the addition of 0.1 mm NAD^+^. The symbols represent the average of *N* = 2 experiments, with a standard deviation of ≤5%.

The reaction mixtures for the activity with UDP‐GlcA (Figs S2–S7) contained 1.0 mm UDP‐GlcA, 100 μm NAD,^+^ and 2.7–81 μm (0.1–3.0 mg·mL^−1^) purified BcUGAepi variant. The reactions for the conversion of UDP‐4‐keto‐pentose (Fig. 3) contained 1.0 mm UDP‐4‐keto‐pentose, 10 mm NADH, and 216 μm (8.0 mg·mL^−1^) of BcUGAepi variant. For measuring the activity with UDP‐4‐keto‐pentose, 27–108 μm (1.0–4.0 mg·mL^−1^) enzyme was used (Figs S8–S12). The reactions for the activity with UDP‐xylose (Figs 3H and S13) contained 1.0 mm UDP‐xylose, 0.1/10 mm NAD,^+^ and 216 μm (8.0 mg·mL^−1^) recombinant BcUGAepi variant. All the reactions (250 μL) were performed in sodium phosphate buffer (50 mm Na_2_HPO_4_, 100 mm NaCl, pH 7.6) and incubated at 23 °C, quenched with methanol (50% (v/v) final concentration) at desired time points, and the precipitated enzyme removed by centrifugation (16 100 *g*, 4 °C, 30 min) prior to HPLC analysis. The initial rates were determined from the linear part of the time course by dividing the slope of the linear regression (mm·min^−1^) by the enzyme concentration (mg·mL^−1^) giving the initial rate in μmol·(min·mg protein)^−1^.

### BcUGAepi reactions for NMR analysis

The reactions of BcUGAepi variants (wild‐type; P85G_Q211G; P85G_T280G; P85G_Q211G_T280G) with UDP‐4‐keto‐pentose were analyzed on ^1^H‐NMR for product identification. The reactions were carried out in D_2_O buffer (50 mm K_2_HPO_4_/KH_2_PO_4_) titrated to pD 7.6, where pD corresponds to the reading of pH meter +0.4. The reaction mixture contained 2.0 mm UDP‐4‐keto‐pentose, 10 mm NADH, and 13.5–189 μm (0.5–7.0 mg·mL^−1^) purified recombinant BcUGAepi variant re‐buffered against D_2_O buffer. The reactions (700 μL each) were incubated at 22 °C for 20 h, the enzymes removed by filtration using 10 kDa cutoff Vivaspin filter tubes, and the supernatant subjected to NMR analysis.

### Analytical methods

#### HPLC—Reactions with UDP‐GlcA

UDP‐GlcA, UDP‐GalA, and NAD^+^/NADH were separated with a Shimadzu Prominence HPLC‐UV system (Shimadzu, Korneuburg, Austria) on a Kinetex C18 column (5 μm, 100 Å, 50 x 4.6 mm) using an isocratic method with 5% acetonitrile and 95% tetrabutylammonium bromide buffer (40 mm TBAB, 20 mm K_2_HPO_4_/KH_2_PO_4_, pH 5.9) as the mobile phase. UDP‐GlcA, UDP‐GalA, NAD,^+^ and NADH were detected by UV at a 262 nm wavelength. The amount of UDP‐GlcA/UDP‐GalA formed was determined based on the relative integrated peak areas and referred to a calibration curve of a commercial standard of UDP‐GlcA.

#### HPLC—Reactions with UDP‐4‐keto‐pentose and UDP‐xylose

UDP‐xylose and UDP‐4‐keto‐pentose were separated with a Shimadzu Prominence HPLC‐UV system on a Kinetex C18 column (5 μm, 100 Å, 150 × 4.6 mm) using an isocratic method with 3% methanol and 97% tetrabutylammonium bromide buffer (40 mm TBAB, 20 mm K_2_HPO_4_/KH_2_PO_4_, pH 5.9) as the mobile phase. UDP‐4‐keto‐pentose and UDP‐xylose were detected by UV at a 262 nm wavelength, and the amount of UDP‐xylose/UDP‐4‐keto‐pentose formed was determined based on the relative integrated peak areas.

#### NMR

The reactions of BcUGAepi variants (wild‐type; P85G_Q211G; P85G_T280G; P85G_Q211G_T280G) with UDP‐4‐keto‐pentose were analyzed on a Varian INOVA 500‐MHz NMR spectrometer (Agilent Technologies, Santa Clara, CA, USA). The VNMRJ 2.2D software was used for the measurements. ^1^H‐NMR spectra (499.98 MHz) were recorded on a 5‐mm indirect detection PFG‐probe with presaturation of the water signal by a shaped pulse. The spectra were analyzed using MestReNova 16.0 (Mestrelab Research, S.L.).

## Results and discussion

### Enzymatic synthesis of UDP‐4‐keto‐pentose

Due to the lack of commercial availability of UDP‐4‐keto‐pentose (UDP‐β‐l‐*threo*‐pentopyranosyl‐4‐ulose), a robust synthesis method was developed. While UDP‐Xyl was readily synthesized from UDP‐GlcA using established protocols [20], the enzymatic production of UDP‐4‐keto‐pentose through existing methods [14] yielded low results in terms of both efficiency and selectivity. The challenge in synthesizing UDP‐4‐keto‐pentose from UDP‐GlcA using the C‐terminal subunit of ArnA (UDP‐GlcA decarboxylase from *Escherichia coli* [21, 22]) stems from ArnA's subsequent reduction of UDP‐4‐keto‐pentose to UDP‐Xyl. The oxidative decarboxylation of UDP‐GlcA by ArnA requires NAD^+^, and the subsequent release of NADH promotes the conversion of UDP‐4‐keto‐pentose to UDP‐Xyl [14]. To address this, we paired the ArnA reaction with a d‐lactate dehydrogenase‐mediated reduction of pyruvate, which efficiently recycles NAD^+^ and eliminates NADH from the reaction mixture (Fig. S14). Applying this reaction cascade, UDP‐GlcA was completely converted to UDP‐4‐keto‐pentose, which was conveniently isolated by a protocol from literature [14].

### Design and preparation of BcUGAepi variants

Wild‐type BcUGAepi displayed only minimal activity when reacted with UDP‐4‐keto‐pentose (1.0 mm) or UDP‐Xyl (1.0 mm) in 10‐fold excess of the coenzyme (NADH or NAD^+^, respectively). To enhance the enzyme's affinity for UDP‐pentose substrates, site‐directed mutagenesis was employed to introduce greater functional flexibility of BcUGAepi, focusing on small alterations in the substrate binding pocket. Comparative analysis of sequence and structure among five UGAepis, three GALEs, three bifunctional GALE/UDP‐xylose 4‐epimerases, and five UDP‐xylose 4‐epimerases, guided by crystal structures (BcUGAepi, GALE) and AlphaFold predictions (UDP‐xylose 4‐epimerase), identified residues presumably influencing substrate specificity (Fig. 2A). Pro85, located within the UDP‐GlcA sugar binding region of BcUGAepi, was identified as a candidate for mutation due to the presence of a highly conserved lysine in all enzymes exhibiting GALE activity and an alanine in UDP‐xylose 4‐epimerases (Fig. 2A,B). Similarly, Gln211 from the UDP‐GlcA uracil binding site was chosen based on the conserved cysteine observed in UDP‐xylose 4‐epimerases, glycine in GALEs, and alanine in bifunctional GALEs (Fig. 2A,B). Additionally, Thr280 in the UDP‐GlcA pyrophosphate region was selected due to a conserved valine in enzymes with UDP‐xylose 4‐epimerase activity and a tyrosine in GALEs, which functions to stabilize the glucose C6‐OH (Fig. 2A,B). To increase the binding site flexibility, we opted to substitute Pro85, Gln211, and Thr280 with glycine, thereby enlarging the space at these positions. Three single variants (P85G; Q211G; T280G), three double variants (P85G_Q211G; P85G_T280G; Q211G_T280G) and the triple variant P85G_Q211G_T280G were successfully prepared by site‐directed mutagenesis (Table S1). The recombinant expression in *E. coli* and subsequent Strep‐tag affinity purification yielded soluble protein in all cases except Q211G_T280G (Fig. S15). The double variant Q211G_T280G evaded soluble expression and was retained in the insoluble fraction after each expression trial (Fig. S15). The soluble‐expressed enzymes were obtained in high purity and an average yield of 13 mg from a liter of culture medium (Fig. S15), except for the Q211G variant, which had an improved expression yield (44 mg·L^−1^) compared to the wild‐type enzyme (~30 mg·L^−1^).

### BcUGAepi variants for UDP‐GlcA epimerization

When offered UDP‐GlcA (1.0 mm) as a substrate, all tested BcUGAepi variants demonstrated epimerization activity, producing UDP‐galacturonic acid (UDP‐GalA) exclusively (Figs S2–S7). The specific activity of the enzymes showed a decrease in response to an increasing number of mutations (Table 1). Specifically, the T280G variant displayed the highest activity at 101 mU·mg^−1^ (Fig. S4), corresponding to a fivefold reduction compared to the wild‐type (500 mU·mg^−1^). Conversely, the triple variant showed only residual activity with UDP‐GlcA (2.0 mU·mg^−1^, Fig. S7), indicating a 250‐fold decline relative to the wild‐type BcUGAepi (Table 1). The single variant Q211G was observed to be the least active at 32.5 mU·mg^−1^ (Table 1, Fig. S3), whereas the P85G variant showed activity of 83 mU·mg^−1^ (Table 1, Fig. S2). Intriguingly, the double variant P85G_Q211G demonstrated ~3.5‐fold higher activity compared to P85G_T280G (Table 1, Figs S5, S6), contrary to expectations based on the performance of the individual variants. This finding illustrates that the combinatorial effects deriving from multiple mutations can often be unpredictable.

**Table 1:** Activities and product ratios of BcUGAepi variants reacted with UDP‐glucuronic acid (UDP‐GlcA) and UDP‐4‐keto‐pentose. UDP‐GlcA: substrate **1**; UDP‐4‐keto‐pentose: substrate **4**. The activities were determined from the linear part of the time course (Figs S2–S7, S8–S12) by dividing the slope of the linear regression (mm·min^−1^) by the enzyme concentration (mg·mL^−1^) giving the initial rate in μmol·(min mg)^−1^, which equals U·mg^−1^. Unless stated otherwise, the ratios are reported after 24 h of reaction time with substrate **1** and after 60 min of reaction time with substrate **4**. UDP‐GalA, UDP‐galacturonic acid; UDP‐Xyl, UDP‐xylose.

Enzyme	Substrate	Activity (mU·mg^−1^)	UDP‐GlcA (**1**, %)	UDP‐GalA (**2**, %)	UDP‐Xyl (**3**, %)	UDP‐4‐keto‐pentose (**4**, %)
Wild‐type	**1**	500	33	67	0	0
P85G	**1**	83	33	67	0	0
Q211G	**1**	32.5	33	67	0	0
T280G	**1**	101	33	67	0	0
P85G_Q211G	**1**	13.8	33	67	0	0
P85G_T280G	**1**	4.0	33	67	0	0
P85G_Q211G_T280G	**1**	2.0	33	67	0	0
Wild‐type	**4**	0.09^b^	–	–	46^a^	54^a^
P85G	**4**	0.10^b^	–	–	64	36
Q211G	**4**	0.04^b^	–	–	34^a^	66^a^
T280G	**4**	0.45^b^	–	–	66^a^	34^a^
P85G_Q211G	**4**	1.7^b^	–	–	86	14
P85G_T280G	**4**	0.9^b^	–	–	82	18
P85G_Q211G_T280G	**4**	6.2^b^	–	–	88	12

^a^ Product ratios are after 12‐h reaction.

^b^ Apparent activity measured from the linear part of the time course after the product burst.

We were additionally interested in whether the mutations of BcUGAepi had changed the substrate specificity of the enzyme so that the GALE substrate UDP‐Glc would be accepted. The wild‐type BcUGAepi does not use UDP‐Glc. However, UDP‐Glc was not reactive with any of the BcUGAepi variants above the detection limit (~5 μm), regardless of the enzyme concentration utilized (up to 10 mg·mL^−1^).

### BcUGAepi variants for UDP‐4‐keto‐pentose reduction

Subsequently, the BcUGAepi variants were assessed using the carboxylate‐lacking UDP‐4‐keto‐pentose substrate (1.0 mm), supplemented with a 10‐fold excess of NADH coenzyme. We observed an incremental increase in the conversion to UDP‐xylose (UDP‐Xyl) that correlated with an increasing number of mutations, indicative of enhanced flexibility of the binding pocket (Fig. 3). While the wild‐type, along with the Q211G and T280G enzymes, catalyzed less than 25% conversion within 30 min of reaction (Fig. 3A–C), both the double and triple variants achieved nearly complete conversion in the same time frame (Fig. 3E–G). The P85G variant displayed ~80% conversion after 30 min (Fig. 3D), demonstrating the substitution of Pro85 as the most beneficial substitution toward the activity increase. This observation is consistent with the knowledge on UDP‐xylose synthase (UXS), which features an alanine residue at a corresponding position and catalyzes the reduction in UDP‐4‐keto‐pentose to UDP‐Xyl (Fig. S16) [20]. Similarly, UDP‐xylose 4‐epimerase, which contains an alanine in the analogous position (Fig. 2), supports the notion that a smaller and structurally less constrained residue than proline in this region enhances activity with UDP‐pentose substrates.

The time‐course characteristics of the UDP‐4‐keto‐pentose reactions showed atypical features across all variants. The reactions displayed “dynamic reversibility,” particularly evident in the P85G variants, where the time courses passed through a kinetic maximum of product release after ~40 min. At longer incubation times up to 12 h, the concentration of UDP‐Xyl product decreased again and the conversion stabilized at approximately 85–90% of UDP‐Xyl for the double and triple mutants (Fig. 3E–G) and 70% for the single mutant (Fig. 3D). Scaled‐up reactions of the double and triple variants analyzed by NMR confirmed UDP‐Xyl as the sole product, with absolutely no evidence of UDP‐L‐arabinose (UDP‐Ara), a C4‐epimer of UDP‐Xyl, which is indistinguishable from UDP‐Xyl on HPLC (Fig. S17). Therefore, the reversibility of reactions involving P85G variants suggests an interconversion between UDP‐4‐keto‐pentose and UDP‐Xyl, indicating that these enzyme variants either cannot perform the necessary rotation in the absence of the carboxylate moiety or fail to stabilize the carboxylate‐lacking intermediate in the correct position for reduction to UDP‐Ara. These observations highlight the critical role of the carboxylate moiety for achieving C4‐epimerization in BcUGAepi.

Furthermore, time‐course analyses of UDP‐4‐keto‐pentose reactions revealed distinctive kinetic behaviors—namely, a rapid initial release of UDP‐Xyl, referred to as “burst,” within the first minute of reaction across all variants (Fig. 3A–G). We then examined the impact of enzyme concentration on this phenomenon, and subsequent reactions were performed at reduced enzyme loading to facilitate activity determination (Table 1; Figs S8–S12). The product burst persisted despite the reduced enzyme concentration, allowing only apparent activity calculation from the after‐burst linear part of the time courses (Table 1; Figs S8–S12). Based on these apparent activities, the variant P85G_Q211G_T280G displayed a ~70‐fold increase in activity for UDP‐4‐keto‐pentose reduction (6.2 mU·mg^−1^; Table 1; Fig. S12) compared to the wild‐type enzyme (0.09 mU·mg^−1^, Table 1, Fig. 3A). Comparison of activities for UDP‐GlcA epimerization and UDP‐4‐keto‐pentose reduction allows for the calculation of a reaction selectivity of each enzyme. This selectivity (activity ratio) was significantly changed between wild‐type enzyme and site‐directed variants (Table 1). The effect was large in particular for the P85G_Q211G_T280G variant that showed an activity ratio of 0.32 whereas that of the wild‐type enzyme was 5.6 × 10^3^. In the other variants, as shown in Table 1, the effect on the activity ratio was smaller than P85G_Q211G_T280G but still substantial.

Detailed analysis of the burst magnitude in respect to enzyme concentration revealed that the burst was either dependent (P85G, P85G_Q211G_T280G) or independent (T280G, P85G_Q211G, P85G_T280G) on the enzyme concentration used (Table 2). The magnitude of the burst, and apparent enzyme activity during the burst, was the most pronounced in the variants containing the P85G substitution (Table 2). The ratio of burst to enzyme concentration ranged between 1.25 and 2.36, and the burst activity between 34–64 mU·mg^−1^, across the P85G‐containing BcUGAepi variants (Table 2). In contrast, the wild‐type, Q211G, and T280G enzymes showed maximum burst activities of 15 mU·mg^−1^ and a maximum burst‐to‐enzyme ratio of 0.61 (Table 2). Based on these results, only the variants containing the P85G mutation exhibited a product burst significantly exceeding the enzyme molarity used (Table 2), and these variants also displayed the highest reduction activity toward UDP‐4‐keto‐pentose (Table 1).

**Table 2:** Product (UDP‐xylose) burst analysis from the reactions of BcUGAepi variants with UDP‐4‐keto‐pentose (1.0 mm) performed in excess of NADH (10 mm). The burst concentration (μm) and activity (mU·mg^−1^) are calculated from the 1‐min time point of the time courses shown in Fig. 3 and Figs S8–S12.

Enzyme	[E] (μm)	[Burst] 1 min (μm)	Burst/enzyme	Burst activity (mU·mg^−1^)
Wild‐type	216	120	0.56	~15
P85G	216	510	2.36	~64
P85G	54	73	1.35	~37
Q211G	216	60	0.28	~8
T280G	216	130	0.60	~16
T280G	108	66	0.61	~16
P85G_Q211G	216	400	1.85	~50
P85G_Q211G	27	47	1.74	~47
P85G_T280G	216	470	2.18	~59
P85G_T280G	27	57	2.11	~57
P85G_Q211G_T280G	216	270	1.25	~34
P85G_Q211G_T280G	27	58	2.15	~58

Considering our previous research on BcUGAepi [6], tight binding/slow release of UDP‐Xyl was postulated as a potential reason underlying the observed product bursts. This hypothesis was supported by experiments in which the addition of UDP‐Xyl (0.1 mm) to the reaction mixture of the wild‐type enzyme led to the abolishment of the product burst, reverting to typical kinetic behavior (Fig. S18). Therefore, it can be deduced that the herein prepared double and triple mutants demonstrate enhanced properties toward UDP‐Xyl binding/release, evidenced by their increased activity toward UDP‐4‐keto‐xylose and its complete conversion to UDP‐Xyl. A burst magnitude that exceeds the enzyme molarity used implies that there exist multiple paths of product release. In a random kinetic mechanism of the enzyme, the ternary product complex (enzyme/NAD^+^/UDP‐xylose) dissociates via two binary complexes, one with NAD^+^ and another with UDP‐xylose. Both binary complexes can recycle the enzyme into a new round of turnover. The flux through the two paths of product dissociation may be different. The distribution of overall flux between the two paths and the extent of rate limitation arising from each determine the observable magnitude of the burst which can thus be larger than unity. A full kinetic characterization of the reduction of UDP‐4‐keto‐pentose by NADH was beyond the scope of this study, but we can conclude from the considerations made above that all BcUGAepi variants harboring the P85G substitution use a complex, probably random mechanism of product release. Evidence that exogenously added UDP‐Xyl suppresses the burst in reactions of the P85G variants supports the suggestion that an abortive ternary complex of enzyme, NADH, and UDP‐Xyl may be formed at steady state when the reaction is performed in the absence of the product. Note that NADH is present in excess in our experiments and given random binding/release of substrates/products it is probable that NADH replaces NAD^+^ in the enzyme complex with UDP‐Xyl. Release of UDP‐Xyl from this abortive complex may be the slowest step of the overall enzymatic process.

We considered the evidence from the reaction time courses of UDP‐4‐keto‐pentose reduction that UDP‐Xyl was slowly reoxidized back to substrate (Fig. 3A‐G). When UDP‐Xyl (1.0 mm) was offered as the sole substrate, the enzyme variants displayed limited oxidation performance, resulting in less than 10% conversion within 12 h (Fig. 3H). The P85G single mutant was an exception, oxidizing approximately 40% of the available UDP‐Xyl to UDP‐4‐keto‐pentose within the first hour of reaction, with no further conversion observed over the subsequent 12 h (Fig. 3H). The addition of NAD^+^ at varying concentrations (0.1 mm or 10 mm) did not affect the final conversion in the P85G variant, whereas a 10‐fold excess of NAD^+^ to UDP‐Xyl significantly improved the conversion from ~5% to ~25% in the wild‐type enzyme (Fig. S13). Importantly, there was no evidence of C‐4 epimerization of UDP‐Xyl in any of the enzymatic reactions analyzed (Fig. 3H). Clearly, the absence of the C‐5 carboxylate group of the substrate made the enzymes lose their native ability to promote stereo‐inversion of the C‐4 by nonstereospecific reduction of the 4‐keto‐intermediate/substrate. Two scenarios seem possible. One is that the 4‐keto‐intermediate/substrate cannot rotate in the enzyme binding pocket when the C‐5 carboxylate group is missing. The other is that the rotational endpoint in the configuration for UDP‐Ara formation lacks suitable stabilization for hydride transfer reduction by NADH due to loss of the original interaction of the C‐5 carboxylate with Arg‐185.

## Conclusions

The current study assessed the role of the C‐5 carboxylate group of the UDP‐GlcA substrate for C‐4 epimerase activity and specificity in the enzyme from *Bacillus cereus*. Site‐directed substitutions were introduced in single, double, and triple variants of BcUGAepi with the aim of increasingly impairing, in an overall fashion, the precise positioning of the substrate for catalysis in the wild‐type enzyme. The substitutions caused a substantial loss of activity, but specificity for conversion of UDP‐GlcA exclusively via C‐4 epimerization was retained. UDP‐β‐l‐*threo*‐pentopyranosyl‐4‐ulose (UDP‐4‐keto‐pentose) was synthesized as a specific probe of the reduction step of the epimerase reaction. The UDP‐4‐keto‐pentose mimics the transient UDP‐4‐keto‐hexuronic acid intermediate of the native epimerase reaction, yet differs from it in the lack of the C‐5 carboxylate group. All enzymes showed activity with UDP‐4‐keto‐pentose and were completely stereospecific for its reduction into UDP‐Xyl. The absence of the C‐5 carboxylate group apparently renders 4‐keto‐intermediate/substrate rotation in the enzyme binding pocket dysfunctional and thus prevents that NADH‐dependent reduction can happen from either face of the C‐4 carbonyl group. The UDP‐4‐keto‐pentose reduction exhibited peculiar burst kinetics, with significant variation of the burst magnitude seen across the different enzymes. A complex, probably random mechanism of product dissociation with UDP‐Xyl release as the rate‐determining step is proposed to explain the kinetic behavior. The P85G_Q211G_T280G variant features the strongest disruption of the overall UDP‐sugar positioning among the enzymes used. It is the most active enzyme for UDP‐4‐keto‐pentose reduction by NADH. Its reaction selectivity (activity ratio of 0.32 for UDP‐GlcA epimerization and UDP‐4‐keto‐pentose reduction) was altered 1.8 × 10^4^‐fold compared to the wild‐type enzyme (ratio: 5.6 × 10^3^). Overall, this study elucidates the structural determinants of substrate specificity of UDP‐glucuronic acid 4‐epimerase in the broader context of related SDR‐type epimerases. The C‐5 carboxylate group is identified as the crucial molecular handle of the substrate to enable the stereo‐inversion of the reactive C‐4 center by the enzyme.

## Conflict of interest

The authors declare no conflict of interest.

## Author contributions

AJEB, BN, design of study; AJEB, LC, experiments; AJEB, analysis of data; AJEB, BN, writing; BN, funding acquisition.

## Peer review

The peer review history for this article is available at https://www.webofscience.com/api/gateway/wos/peer‐review/10.1002/1873‐3468.70070.

## Supporting information


**Fig. S1.** Reaction scheme for the interconversion of UDP‐Glc and UDP‐Gal catalyzed by GALE.
**Fig. S2.** Time course of BcUGAepi_P85G catalyzed reaction with UDP‐GlcA as a substrate.
**Fig. S3.** Time course of BcUGAepi_Q211G catalyzed reaction with UDP‐GlcA as a substrate.
**Fig. S4.** Time course of BcUGAepi_T280G catalyzed reaction with UDP‐GlcA as a substrate.
**Fig. S5.** Time course of BcUGAepi_P85G_Q211G catalyzed reaction with UDP‐GlcA as a substrate.
**Fig. S6.** Time course of BcUGAepi_P85G_T280G catalyzed reaction with UDP‐GlcA as a substrate.
**Fig. S7.** Time course of BcUGAepi_P85G_Q211G_T280G catalyzed reaction with UDP‐GlcA as a substrate.
**Fig. S8.** Time course of BcUGAepi_P85G catalyzed reaction with UDP‐4‐keto‐pentose as a substrate for activity calculation.
**Fig. S9.** Time course of BcUGAepi_T280G catalyzed reaction with UDP‐4‐keto‐pentose as a substrate for activity calculation.
**Fig. S10.** Time course of BcUGAepi_P85G_Q211G catalyzed reaction with UDP‐4‐keto‐pentose as a substrate for activity calculation.
**Fig. S11.** Time course of BcUGAepi_P85G_T280G catalyzed reaction with UDP‐4‐keto‐pentose as a substrate for activity calculation.
**Fig. S12.** Time course of BcUGAepi_P85G_Q211G_T280G catalyzed reaction with UDP‐4‐keto‐pentose as a substrate for activity calculation.
**Fig. S13.** Overlay of the brief time courses of BcUGAepi wild‐type and P85G variants reacted with UDP‐xylose in the presence of different NAD^+^ concentrations (0.1 or 10 mm).
**Fig. S14.** Reaction scheme for the production of UDP‐4‐keto‐pentose from UDP‐GlcA catalyzed by ArnA, including the NAD^+^ regeneration system provided by d‐lactate dehydrogenase (D‐LDH).
**Fig. S15.** SDS/polyacrylamide gels from the Strep‐tag purifications of BcUGAepi variants (∼37 kDa) and after B‐PER treatment of Q211G_T280G variant.
**Fig. S16.** Active site close‐ups of BcUGAepi (green; PDB: 6Z73; yellow carbons UDP‐GlcA, light gray carbons NAD^+^) and UXS (cyan; PDB: 2B69; orange carbons UDP‐GlcA, dark gray carbons NAD^+^) substrate complexes showing the location of Pro85 (BcUGAepi) and Ala79 (UXS).
**Table S1.** Sequences of DNA oligonucleotide primers used for the mutagenesis in BcUGAepi.

## Data Availability

The data that support the findings of this study are available from the corresponding author [bernd.nidetzky@tugraz.at] upon reasonable request.
