# Supplementary material for: The carboxylate “gripper” of the substrate is critical for C‐4 stereo‐inversion by UDP‐glucuronic acid 4‐epimerase
Source: FEBS Lett. 2025 May 16;599(15):2190–200. doi: 10.1002/1873-3468.70070 (PMC12338863; doi:10.1002/1873-3468.70070)
Supplement: Supplementary file 1 — Fig. S1. Reaction scheme for the interconversion of UDP‐Glc and UDP‐Gal catalyzed by GALE. Fig. S2. Time course of BcUGAepi_P85G catalyzed reaction with UDP‐GlcA as a substrate. Fig. S3. Time course of BcUGAepi_Q211G catalyzed reaction with UDP‐GlcA as a substrate. Fig. S4. Time course of BcUGAepi_T280G catalyzed reaction with UDP‐GlcA as a substrate. Fig. S5. Time course of BcUGAepi_P85G_Q211G catalyzed reaction with UDP‐GlcA as a substrate. Fig. S6. Time course of BcUGAepi_P85G_T280G catalyzed reaction with UDP‐GlcA as a substrate. Fig. S7. Time course of BcUGAepi_P85G_Q211G_T280G catalyzed reaction with UDP‐GlcA as a substrate. Fig. S8. Time course of BcUGAepi_P85G catalyzed reaction with UDP‐4‐keto‐pentose as a substrate for activity calculation. Fig. S9. Time course of BcUGAepi_T280G catalyzed reaction with UDP‐4‐keto‐pentose as a substrate for activity calculation. Fig. S10. Time course of BcUGAepi_P85G_Q211G catalyzed reaction with UDP‐4‐keto‐pentose as a substrate for activity calculation. Fig. S11. Time course of BcUGAepi_P85G_T280G catalyzed reaction with UDP‐4‐keto‐pentose as a substrate for activity calculation. Fig. S12. Time course of BcUGAepi_P85G_Q211G_T280G catalyzed reaction with UDP‐4‐keto‐pentose as a substrate for activity calculation. Fig. S13. Overlay of the brief time courses of BcUGAepi wild‐type and P85G variants reacted with UDP‐xylose in the presence of different NAD+ concentrations (0.1 or 10 mm). Fig. S14. Reaction scheme for the production of UDP‐4‐keto‐pentose from UDP‐GlcA catalyzed by ArnA, including the NAD+ regeneration system provided by d‐lactate dehydrogenase (D‐LDH). Fig. S15. SDS/polyacrylamide gels from the Strep‐tag purifications of BcUGAepi variants (∼37 kDa) and after B‐PER treatment of Q211G_T280G variant. Fig. S16. Active site close‐ups of BcUGAepi (green; PDB: 6Z73; yellow carbons UDP‐GlcA, light gray carbons NAD+) and UXS (cyan; PDB: 2B69; orange carbons UDP‐GlcA, dark gray carbons NAD+) substrate complexes showing th [file FEB2-599-2190-s001.docx]

**SUPPLEMENTARY INFORMATION**

**The carboxylate ‘gripper’ of the substrate is critical for C-4 stereo-inversion by UDP-glucuronic acid 4-epimerase**

**Annika J. E. Borg^[a]^, Laura De Cnop^[a]^ and Bernd Nidetzky^[a,b]*^**

[a] Institute of Biotechnology and Biochemical Engineering, Graz University of Technology, NAWI Graz, Petersgasse 12, 8010 Graz, Austria

[b] Austrian Centre of Industrial Biotechnology (acib), Krenngasse 37, 8010 Graz, Austria

* Corresponding author (B.N., bernd.nidetzky@tugraz.at)

Keywords: SDR, short-chain dehydrogenase/reductase, epimerase, UDP-glucuronic acid, stereo-inversion, rotation, UDP-4-keto-pentose, reductase, UDP-xylose

Abbreviations: SDR, short-chain dehydrogenase/reductase; UGAepi, UDP-glucuronic acid 4-epimerase; GALE, UDP-galactose 4-epimerase; UDP-GlcA, UDP-glucuronic acid; UDP-GalA, UDP-galacturonic acid; UDP-Xyl, UDP-xylose

| **Name** | **Mutation** | **DNA primer sequence (5’-3’)** |
| --- | --- | --- |
| P85G_fw  P85G_rv | P85G  P85G | CATCTGGCGGCGATCGGTGGCGTTCGTACCAG CTGGTACGAACGCCACCGATCGCCGCCAGATG |
| Q211G_fw  Q211G_rv | Q211G  Q211G | CGGTGACGGCACCGGTACCCGTGATTTTACCTATATTG CAATATAGGTAAAATCACGGGTACCGGTGCCGTCACCG |
| T280G_fw  T280G_rv | T280G  T280G | CGAGCCGAAACAGGGTTGGGCGGATATTAG CTAATATCCGCCCAACCCTGTTTCGGCTCG |

**Table S1**. The sequences of DNA oligonucleotide primers used for the mutagenesis in BcUGAepi.

**Figure S1**. Reaction scheme for the interconversion of UDP-Glc and UDP-Gal catalyzed by GALE [1].


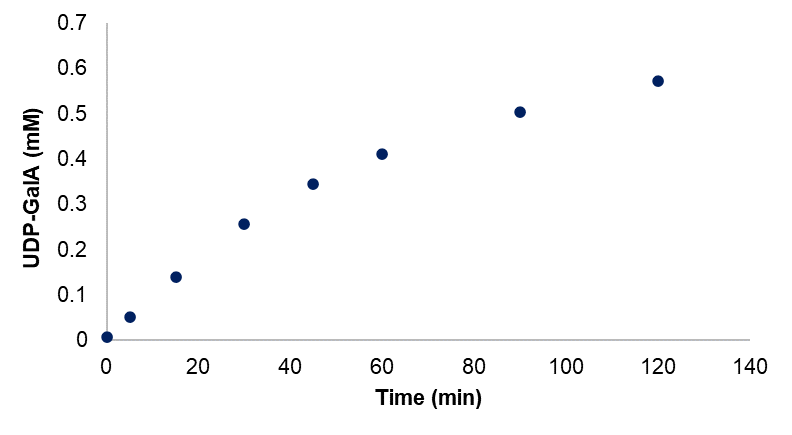


**Figure S2.** Time course of BcUGAepi_P85G catalyzed reaction with UDP-GlcA as a substrate. The reaction was performed with 1.0 mM UDP-GlcA, 100 µM NAD^+^ and 2.7 µM (0.1 mg/mL) purified recombinant BcUGAepi_P85G in sodium phosphate buffer (50 mM Na_2_HPO_4_, 100 mM NaCl, pH 7.6) in final volume of 250 µl. The activity of P85G variant (83 mU/mg) was calculated from the initial velocity (linear part, 0-30 min) of the time course. The symbols represent the average of *N* = 2 experiments, with a standard deviation of ≤5%.


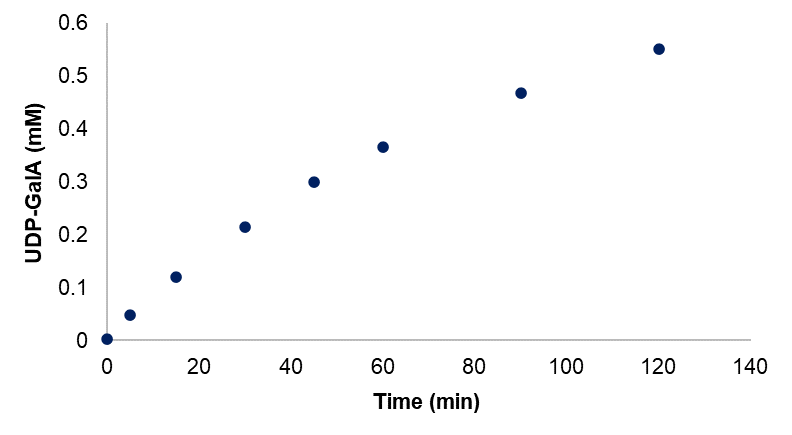


**Figure S3.** Time course of BcUGAepi_Q211G catalyzed reaction with UDP-GlcA as a substrate. The reaction was performed with 1.0 mM UDP-GlcA, 100 µM NAD^+^ and 5.4 µM (0.2 mg/mL) purified recombinant BcUGAepi_Q211G in sodium phosphate buffer (50 mM Na_2_HPO_4_, 100 mM NaCl, pH 7.6) in final volume of 250 µl. The activity of Q211G variant (32.5 mU/mg) was calculated from the initial velocity (linear part, 0-45 min) of the time course. The symbols represent the average of *N* = 2 experiments, with a standard deviation of ≤5%.

**
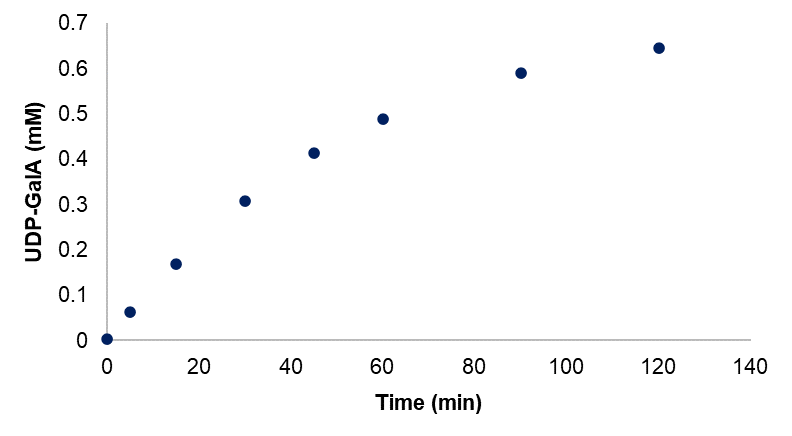
**

**Figure S4.** Time course of BcUGAepi_T280G catalyzed reaction with UDP-GlcA as a substrate. The reaction was performed with 1.0 mM UDP-GlcA, 100 µM NAD^+^ and 2.7 µM (0.1 mg/mL) purified recombinant BcUGAepi_T280G in sodium phosphate buffer (50 mM Na_2_HPO_4_, 100 mM NaCl, pH 7.6) in final volume of 250 µl. The activity of T280G variant (101 mU/mg) was calculated from the initial velocity (linear part, 0-30 min) of the time course. The symbols represent the average of *N* = 2 experiments, with a standard deviation of ≤5%.

**
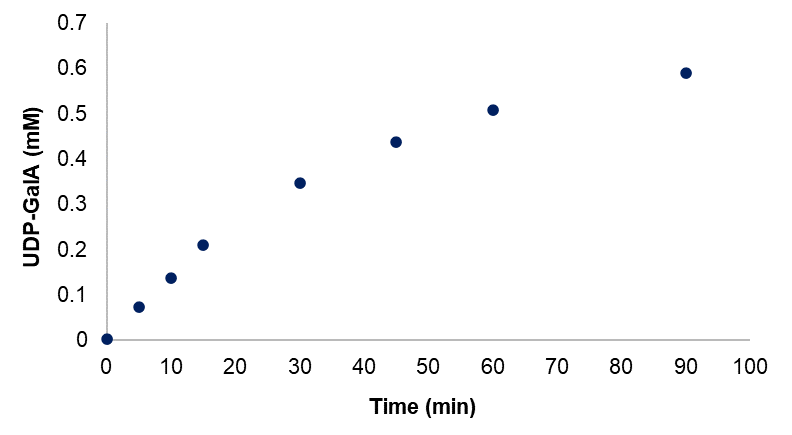
**

**Figure S5.** Time course of BcUGAepi_P85G_Q211G catalyzed reaction with UDP-GlcA as a substrate. The reaction was performed with 1.0 mM UDP-GlcA, 100 µM NAD^+^ and 27 µM (1.0 mg/mL) purified recombinant BcUGAepi_P85G_Q211G in sodium phosphate buffer (50 mM Na_2_HPO_4_, 100 mM NaCl, pH 7.6) in final volume of 250 µl. The activity of P85G_Q211G variant (13.8 mU/mg) was calculated from the initial velocity (linear part, 0-15 min) of the time course. The symbols represent the average of *N* = 2 experiments, with a standard deviation of ≤5%.

**
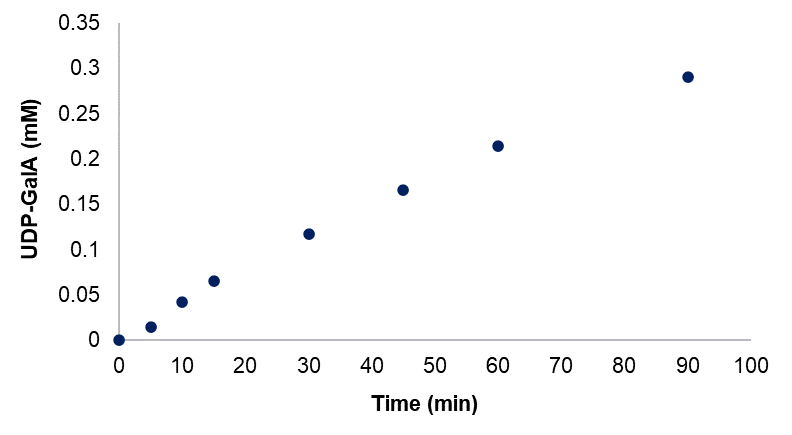
**

**Figure S6.** Time course of BcUGAepi_P85G_T280G catalyzed reaction with UDP-GlcA as a substrate. The reaction was performed with 1.0 mM UDP-GlcA, 100 µM NAD^+^ and 27 µM (1.0 mg/mL) purified recombinant BcUGAepi_P85G_T280G in sodium phosphate buffer (50 mM Na_2_HPO_4_, 100 mM NaCl, pH 7.6) in final volume of 250 µl. The activity of P85G_T280G variant (4.0 mU/mg) was calculated from the initial velocity (linear part, 0-30 min) of the time course. The symbols represent the average of *N* = 2 experiments, with a standard deviation of ≤5%.

**
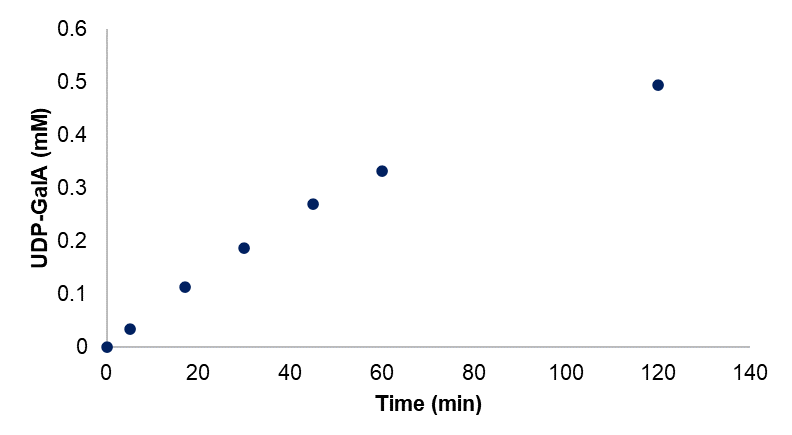
**

**Figure S7**. Time course of BcUGAepi_P85G_Q211G_T280G catalyzed reaction with UDP-GlcA as a substrate. The reaction was performed with 1.0 mM UDP-GlcA, 100 µM NAD^+^ and 81 µM (3.0 mg/mL) purified recombinant BcUGAepi_P85G_Q211G_T280G in sodium phosphate buffer (50 mM Na_2_HPO_4_, 100 mM NaCl, pH 7.6) in final volume of 250 µl. The activity of P85G_Q211G_T280G variant (2.0 mU/mg) was calculated from the initial velocity (linear part, 0-45 min) of the time course. The symbols represent the average of *N* = 2 experiments, with a standard deviation of ≤5%.

**
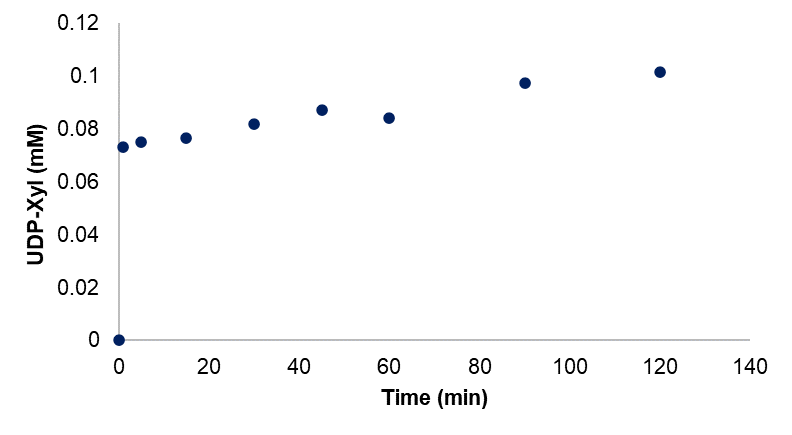
**

**Figure S8.** Time course of BcUGAepi_P85G catalyzed reaction with UDP-4-keto-pentose as a substrate for activity calculation. The reaction was performed with 1.0 mM UDP-4-keto-pentose, 10 mM NADH and 54 µM (2.0 mg/mL) purified recombinant BcUGAepi_P85G in sodium phosphate buffer (50 mM Na_2_HPO_4_, 100 mM NaCl, pH 7.6) in final volume of 250 µl. The activity of BcUGAepi_P85G (0.1 mU/mg) was calculated from the linear part (1-120 min) of the time course. The symbols represent the average of *N* = 2 experiments, with a standard deviation of ≤5%.

**
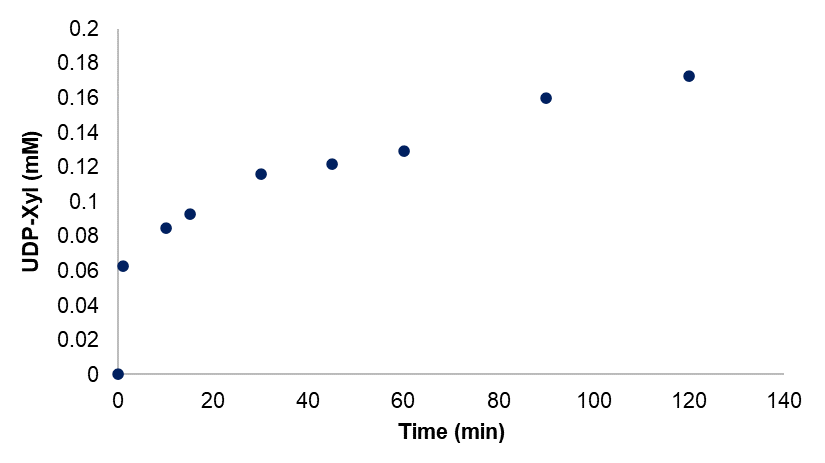
**

**Figure S9.** Time course of BcUGAepi_T280G catalyzed reaction with UDP-4-keto-pentose as a substrate for activity calculation. The reaction was performed with 1.0 mM UDP-4-keto-pentose, 10 mM NADH and 108 µM (4.0 mg/mL) purified recombinant BcUGAepi_T280G in sodium phosphate buffer (50 mM Na_2_HPO_4_, 100 mM NaCl, pH 7.6) in final volume of 250 µl. The activity of BcUGAepi_T280G (0.45 mU/mg) was calculated from the linear part (1-30 min) of the time course. The symbols represent the average of *N* = 2 experiments, with a standard deviation of ≤5%.

**
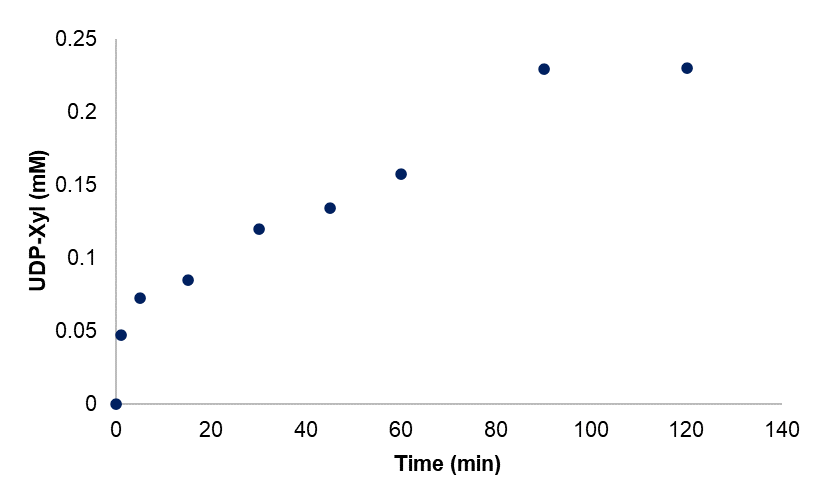
**

**Figure S10.** Time course of BcUGAepi_P85G_Q211G catalyzed reaction with UDP-4-keto-pentose as a substrate for activity calculation. The reaction was performed with 1.0 mM UDP-4-keto-pentose, 10 mM NADH and 27 µM (1.0 mg/mL) purified recombinant BcUGAepi_P85G_Q211G in sodium phosphate buffer (50 mM Na_2_HPO_4_, 100 mM NaCl, pH 7.6) in final volume of 250 µl. The activity of BcUGAepi_P85G_Q211G (1.7 mU/mg) was calculated from the linear part (1-60 min) of the time course. The symbols represent the average of *N* = 2 experiments, with a standard deviation of ≤5%.

**
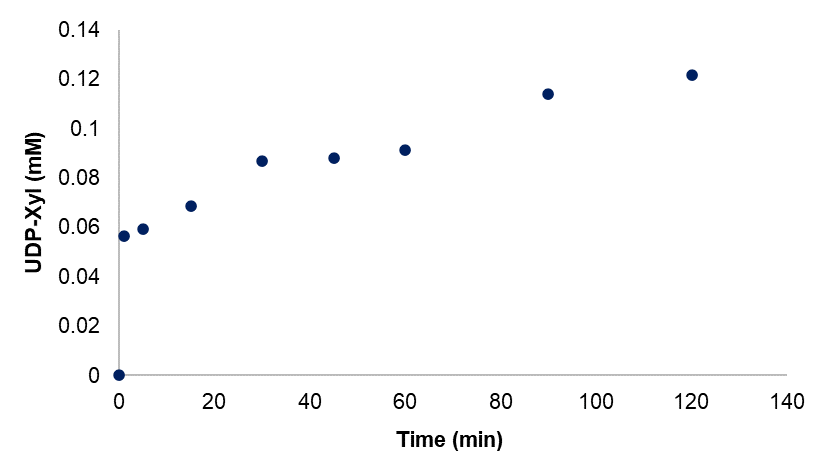
**

**Figure S11.** Time course of BcUGAepi_P85G_T280G catalyzed reaction with UDP-4-keto-pentose as a substrate for activity calculation. The reaction was performed with 1.0 mM UDP-4-keto-pentose, 10 mM NADH and 27 µM (1.0 mg/mL) purified recombinant BcUGAepi_P85G_T280G in sodium phosphate buffer (50 mM Na_2_HPO_4_, 100 mM NaCl, pH 7.6) in final volume of 250 µl. The activity of BcUGAepi_P85G_T280G (0.9 mU/mg) was calculated from the linear part (1-15 min) of the time course. The symbols represent the average of *N* = 2 experiments, with a standard deviation of ≤5%.

**
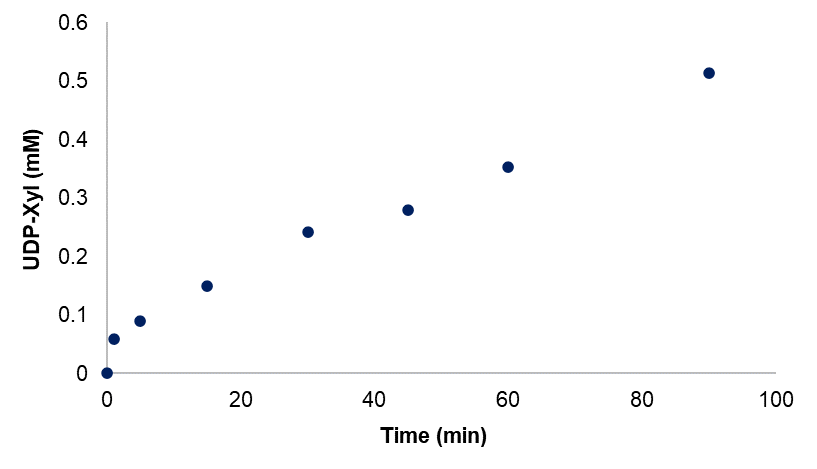
**

**Figure S12.** Time course of BcUGAepi_P85G_Q211G_T280G catalyzed reaction with UDP-4-keto-pentose as a substrate for activity calculation. The reaction was performed with 1.0 mM UDP-4-keto-pentose, 10 mM NADH and 27 µM (1.0 mg/mL) purified recombinant BcUGAepi_P85G_Q211G_T280G in sodium phosphate buffer (50 mM Na_2_HPO_4_, 100 mM NaCl, pH 7.6) in final volume of 250 µl. The activity of BcUGAepi_P85G_Q211G_T280G (6.2 mU/mg) was calculated from the linear part (1-30 min) of the time course. The symbols represent the average of *N* = 2 experiments, with a standard deviation of ≤5%.

**Figure S13.** Overlay of the brief time courses of BcUGAepi wild type and P85G variants reacted with UDP-xylose in the presence of different NAD^+^ concentrations (0.1 or 10 mM). Each reaction was performed with 1.0 mM UDP-xylose, 0.1/10 mM NAD^+^ and 216 µM (8.0 mg/mL) purified recombinant BcUGAepi wild type or P85G variant in sodium phosphate buffer (50 mM Na_2_HPO_4_, 100 mM NaCl, pH 7.6) in final volume of 250 µl. The symbols represent the average of *N* = 2 experiments with 0.1 mM NAD^+^ (with a standard deviation of ≤5%). Reactions with 10 mM NAD^+^ were performed once (*N* = 1).

**Figure S14**. Reaction scheme for the production of UDP-4-keto-pentose from UDP-GlcA catalyzed by ArnA [2], including the NAD^+^ regeneration system provided by d-lactate dehydrogenase (D-LDH).

*
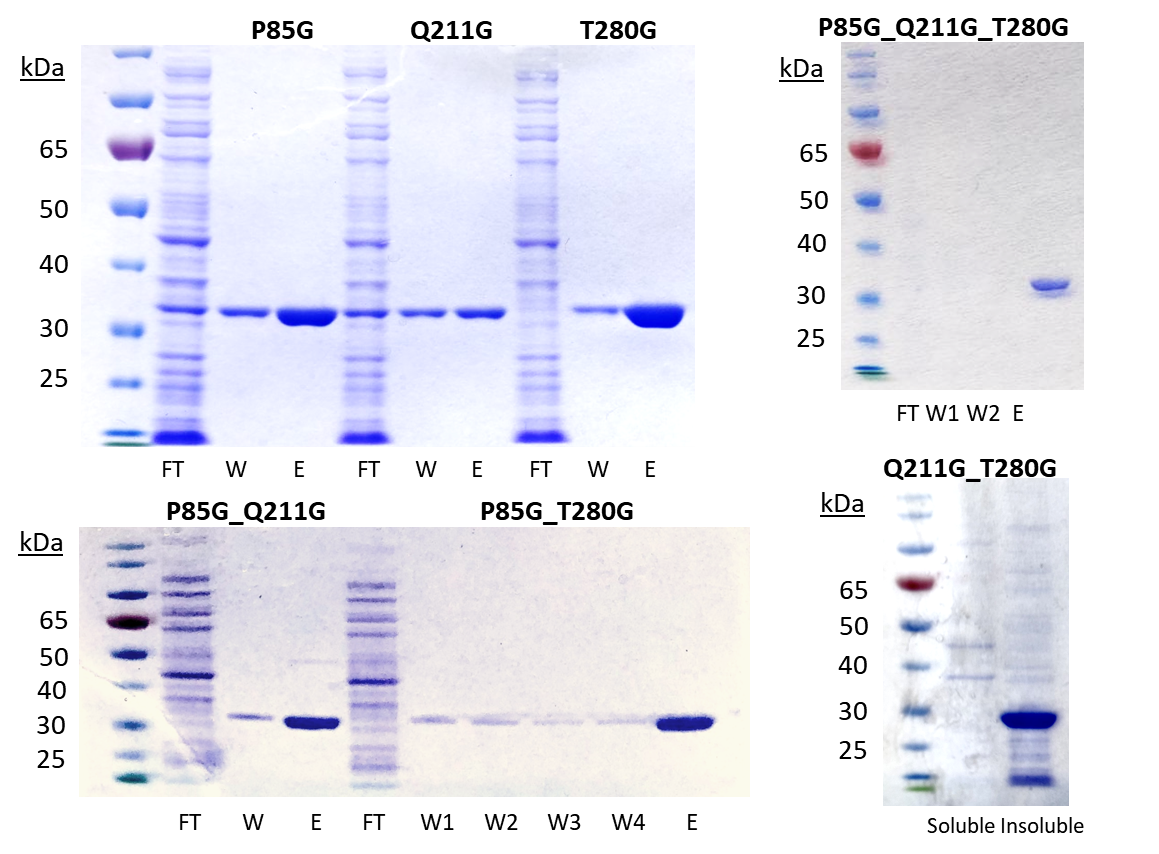
*

**Figure S15**. SDS-polyacrylamide gels from the Strep-tag purifications of BcUGAepi variants (⁓37 kDa) and after B-PER treatment of Q211G_T280G variant. FT = flow through, unbound proteins; W = washing fraction; E = elution fraction.


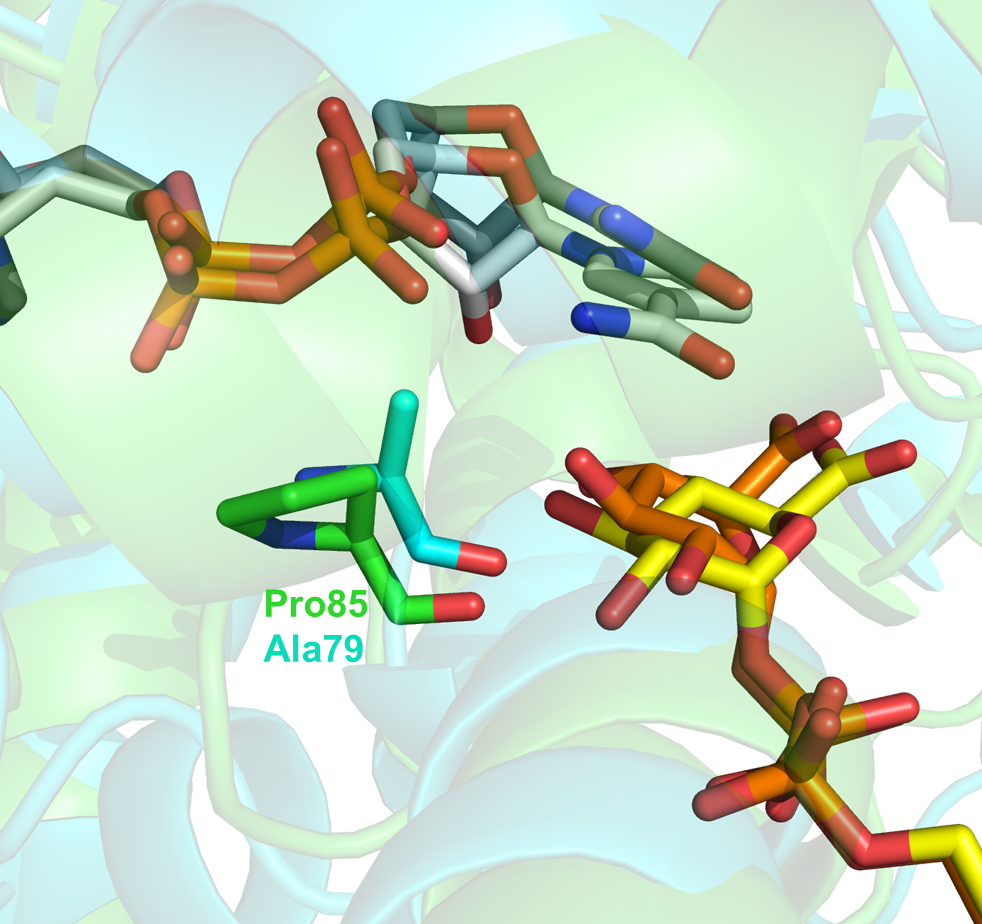


**Figure S16.** Active site close-ups of BcUGAepi (green; PDB: 6Z73; yellow carbons UDP-GlcA, light gray carbons NAD^+^) [3] and UXS (cyan; PDB: 2B69; orange carbons UDP-GlcA, dark gray carbons NAD^+^) [4] substrate complexes showing the location of Pro85 (BcUGAepi) and Ala79 (UXS). The structural overlay was generated using PyMOL v4.6.


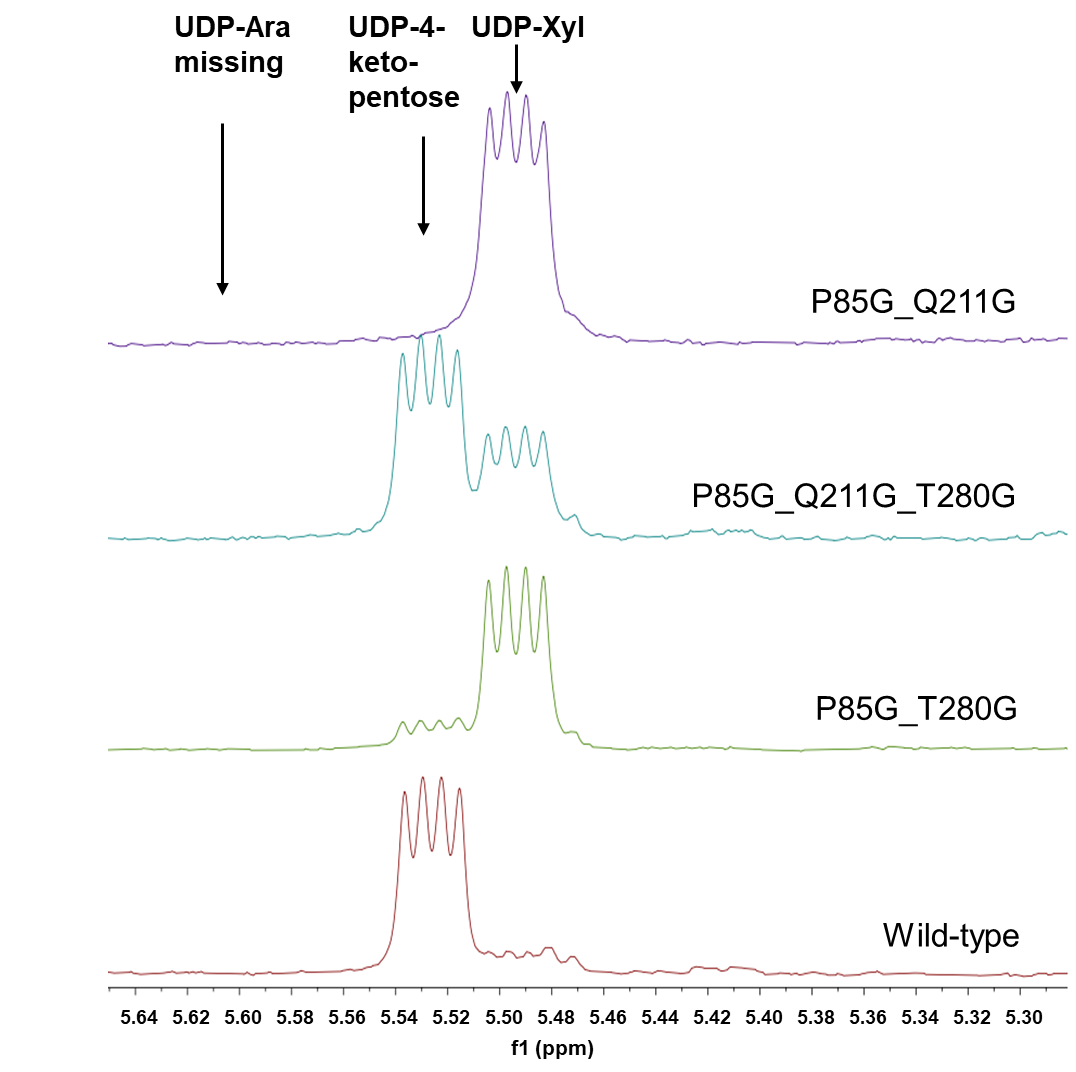


**Figure S17.** ^1^H-NMR (500 MHz, D_2_O) analysis of the reaction mixtures of BcUGAepi variants reacted with UDP-4-keto-pentose. Only the close-up of the anomeric region is shown. The low conversion rate in the P85G_Q211G_T280G reaction is due to the activity decrease in D_2_O and low enzyme concentration used in the reaction. UDP-xylose, 5.49 ppm; UDP-4-keto-pentose, 5.53 ppm. If UDP-arabinose was present, a signal would be seen at 5.65 ppm.

**Figure S18**. Time course of BcUGAepi wild type reacted with UDP-4-keto-pentose (1.0 mM) in presence of UDP-Xyl (0.1 mM). The reaction contained 1.0 mM UDP-4-keto-pentose, 0.1 mM UDP-Xyl, 10 mM NADH and 216 µM (8.0 mg/mL) purified BcUGAepi wild-type in sodium phosphate buffer (50 mM Na_2_HPO_4_, 100 mM NaCl, pH 7.6) in final volume of 250 µl. The symbols represent the average of *N* = 2 experiments, with a standard deviation of ≤5%.

1. Thoden JB, Holden HM: **Dramatic differences in the binding of UDP-galactose and UDP-glucose to UDP-galactose 4-epimerase from *Escherichia coli***. *Biochemistry* 1998, **37**:11469–11477.

2. Williams GJ, Breazeale SD, Raetz CRH, Naismith JH: **Structure and function of both domains of ArnA, a dual function decarboxylase and a formyltransferase, involved in 4-amino-4-deoxy-l-arabinose biosynthesis**. *J Biol Chem* 2005, **280**:23000–23008.

3. Iacovino LG, Savino S, Borg AJE, Binda C, Nidetzky B, Mattevi A: **Crystallographic snapshots of UDP-glucuronic acid 4-epimerase ligand binding, rotation, and reduction**. *J Biol Chem* 2020, **295**:12461–12473.

4. Eixelsberger T, Sykora S, Egger S, Brunsteiner M, Kavanagh KL, Oppermann U, Brecker L, Nidetzky B: **Structure and mechanism of human UDP-xylose synthase: evidence for a promoting role of sugar ring distortion in a three-step catalytic conversion of UDP-glucuronic acid.** *J Biol Chem* 2012, **287**:31349–58.
